# Natural Supplements for H1N1 Influenza: Retrospective Observational Infodemiology Study of Information and Search Activity on the Internet

**DOI:** 10.2196/jmir.1722

**Published:** 2011-05-10

**Authors:** Shawndra Hill, Jun Mao, Lyle Ungar, Sean Hennessy, Charles E Leonard, John Holmes

**Affiliations:** ^4^Department of Biostatistics and EpidemiologySchool of MedicineUniversity of PennsylvaniaPhiladelphia, PAUnited States; ^3^Department of Computer ScienceSchool of Engineering and Applied ScienceUniversity of PennsylvaniaPhiladelphia, PAUnited States; ^2^Department of Family Medicine and Community HealthSchool of MedicineUniversity of PennsylvaniaPhiladelphia, PAUnited States; ^1^Operations and Information ManagementThe Wharton SchoolUniversity of PennsylvaniaPhiladelphia,PAUnited States

**Keywords:** Internet search, pandemic, herbal supplements, H1N1 influenza

## Abstract

**Background:**

As the incidence of H1N1 increases, the lay public may turn to the Internet for information about natural supplements for prevention and treatment.

**Objective:**

Our objective was to identify and characterize websites that provide information about herbal and natural supplements with information about H1N1 and to examine trends in the public’s behavior in searching for information about supplement use in preventing or treating H1N1.

**Methods:**

This was a retrospective observational infodemiology study of indexed websites and Internet search activity over the period January 1, 2009, through November 15, 2009. The setting is the Internet as indexed by Google with aggregated Internet user data. The main outcome measures were the frequency of “hits” or webpages containing terms relating to natural supplements co-occurring with H1N1/swine flu, terms relating to natural supplements co-occurring with H1N1/swine flu proportional to all terms relating to natural supplements, webpage rank, webpage entropy, and temporal trend in search activity.

**Results:**

A large number of websites support information about supplements and H1N1. The supplement with the highest proportion of H1N1/swine flu information was a homeopathic remedy known as Oscillococcinum that has no known side effects; supplements with the next highest proportions have known side effects and interactions. Webpages with both supplement and H1N1/swine flu information were less likely to be medically curated or authoritative. Search activity for supplements was temporally related to H1N1/swine flu-related news reports and events.

**Conclusions:**

The prevalence of nonauthoritative webpages with information about supplements in the context of H1N1/swine flu and the increasing number of searches for these pages suggest that the public is interested in alternatives to traditional prevention and treatment of H1N1. The quality of this information is often questionable and clinicians should be cognizant that patients may be at risk of adverse events associated with the use of supplements for H1N1.

## Introduction

The 2009 pandemic of influenza A (H1N1) has affected at least 199 countries, with 482,300 confirmed cases and more than 6070 deaths worldwide as of November 1, 2009 [1]. These numbers are underestimates since most countries have stopped reporting cases, and mild cases are difficult to track. Although H1N1 influenza is no more severe than typical influenzas, its widespread dispersal will result in enormous human and economic cost to society.

The Web is a source for information on almost everything, and people search the Web from all over the world. So it is no surprise that when faced with emerging diseases where treatments are few and treatment distribution is limited, individuals often search the Internet for information, for example, on H1N1 prevention or treatment. In addition, new social media technologies such as Twitter enable people to follow reputable sources to get information about H1N1 as well as communicate about epidemics with peers [2]. This is particularly true for vitamin, mineral, and herbal supplement therapies, partly because of the wide use of such modalities [2,4,5]. In this paper, we are not only interested in search queries and their link to epidemics [3,6,7], but also in which webpages people land on after they search for vitamin, mineral, and herbal supplement treatments.

The goal of this study was to examine searches of the Internet for natural products used to prevent or treat H1N1. Although the definition of natural products may be different in different contexts, for this paper we focused on natural products that are not made of synthetic compounds and can be purchased over the counter. More specifically, we are interested in those that can be consumed orally, such as herbs, vitamins, natural supplements, or homeopathic products, since these products have a greater potential to cause harm compared with topical products or nonpharmacological therapies (eg, yoga and meditation) when used in conjunction with prescribed medications. For the purposes of this paper, we call these products *supplements*. 

It has been shown that the information displayed about medical supplement products on the Web is often inaccurate or even fraudulent [8]. One study found that 25% of 150 websites on supplement information contained information that could lead to direct physical harm if acted upon [8]. Another study found that medical information websites containing information on supplements were more likely to contain inaccurate information [9]. Furthermore, some supplement products purchased over the Internet contain heavy metals that could negatively affect health [10]. Physicians are often unaware of the herbal and other supplements that their patients are taking, as patients rarely disclose such therapies to their physicians [11]. Despite the potential pitfalls of using online user-contributed content for health surveillance, it is hard to ignore the potential to harness the Web for surveillance [12], and there have been recent discussions in the literature on "infodemiology" frameworks for measuring the value of different types on online digital information [13].

To our knowledge, no published study has evaluated how the rise in the H1N1 epidemic relates to Internet searches regarding supplements. However, an understanding of this issue is critical from at least two perspectives. In terms of public health, monitoring public H1N1-related search activities may provide federal agencies with estimates of the prevalence of such information-seeking behavior. Improved awareness may prompt policy development to better regulate the quality of such publically disseminated information. In terms of clinical practice, clinicians would benefit from knowing what common herbs and natural products their patients are learning about to deal with H1N1 influenza. Such information can improve patient-physician communication to promote evidence-based supplement use.

The specific objectives of our investigation were twofold: to identify and characterize websites that provide information about H1N1 alongside information on supplements and to examine trends in the public’s information-seeking behavior with respect to supplement use for preventing or treating H1N1. To meet these objectives, we sought to answer the following research questions: (1) What is the availability of information about supplements and H1N1? (2) What supplements does the public search for, and how do search patterns change over time? (3) What types of websites provide information about supplements and H1N1?

## Methods

### Source of Data

We used three sources to compile a list of supplements for this investigation: drug information from the National Library of Medicine [14], the HubPage on homeopathic remedies [15], and a list of supplements compiled by one of the authors (JM). This list was based on a qualitative and exhaustive Internet search using the key words *herb*, *natural*, *flu*, *cold*, *swine flu*, and *H1N1* and was conducted on October 29, 2009. A controlled vocabulary was created by merging these lists, removing duplicates, and resolving abbreviations and synonymy. We used both common and botanical names where appropriate. The final version of the controlled vocabulary contained 145 search terms and is available as Multimedia Appendix 1.

### Search Method

We used a simple computer program (available on request) to perform automated Internet searches with Google, which was used because it is the most frequently utilized search engine. Our program performed a search using each item in the controlled vocabulary described above in combination with both *H1N1* and *swine flu* separately as a single search term. Examples of the resultant single search terms are *H1N1 Vitamin A* and *swine flu Vitamin A*. The results of the search for each term were written to a machine-readable file as the term with the associated number of indexed pages (“hits”). This search was performed on November 15, 2009. We used our code instead of publicly available software because our queries were straightforward to perform and we could run in batch mode, in parallel, on our university grid computer.

### Metrics and Analysis

We calculated several metrics for this investigation. The first metric was F_s__upplement_, defined as the frequency of hits counted separately for each supplement together with H1N1. Thus, F_s__upplement_ reflects the prevalence of information about supplements co-occurring with H1N1 on the Internet, regardless of the context, including advertising, mentions on discussion boards or other social media, or health-related contexts.

The second metric is P_s__upplement_, defined as the proportion of total hits for a given supplement that also include H1N1 on the webpage:


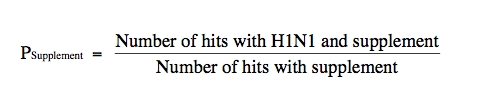


The third metric we used is *page rank*, a metric calculated by Google that indicates the popularity and importance of a webpage [16]. The rank of a webpage is based on page rank and the number of “incoming” links (pages from which users have navigated to arrive at a webpage) and the number of hits the page receives over time. Links to webpages are listed by order of page rank, after sponsored links, on the results of queries returned by Google. Although most queries return multiple pages of results, we focused only on the first page of search results, since most users select links from this page without exploring the others [17]. We ordered page ranks as a range from 1 (most relevant) to 10 (least relevant).

Fourth, we evaluated the variability of webpages returned by each query using an information theoretic metric, *Shannon’s entropy*:


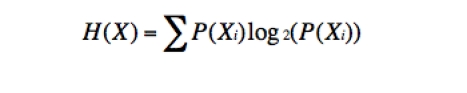


where entropy, or H(X), is the uncertainty of variable X, P(X_i_) is the probability of observing category *i* in the data, where *i* represents a specific webpage in our data, for example, the webpage for the Centers for Disease Control and Prevention (CDC). In our context, entropy reflects the number of different webpages that are returned in the top slot by queries on Google. We compared two query types: queries for the supplement alone and queries with each supplement plus H1N1. We use entropy to measure how stable the webpage returned in the top slot is; for example, do we always get the CDC’s webpage in the first slot when submitting our queries, or, instead, do we get many different webpages.

As a fifth metric, we used Google Trends [16] to investigate temporal trends in search behavior. This service samples Google Web-searching activity and reports the volume of searches for a specific term relative to all Google searches over time. We investigated the trends associated with the most frequently searched-for supplements (based on P_Supplement_) together with the term *flu*. We were restricted to this term because Google Trends did not detect sufficient search activity using the more descriptive terms *swine flu* or *H1N1* combined with searches for supplements. We don’t believe this restriction limited our study because we compared Google trends curves for *flu* and *swine flu* and they have the very same shape over time with the same peaks at the height of the outbreak.

The observation period was from January 1, 2009, through November 15, 2009. The search volumes were compared with specific news events that occurred during this time period to examine the effect of these events on search behavior. We measured the probability of hits returned by the queries for supplement plus H1N1 by domain or function (such as .gov, .com, news, etc.). Finally, we identified the side effect and interaction profiles of the 20 supplements with the highest P_Supplement_ values.

## Results

### Availability of Information on the Web.

There is a significant amount of information about the H1N1 pandemic on various webpages in the form of health alerts by governments, news stories, advertisements, and user-generated content on blogs, discussion boards, and popular social networking portals like Facebook and Twitter. Table 1 displays the supplements found on the 20 pages that most frequently included both a supplement from the controlled vocabulary and H1N1, ranked by F_supplement_. Table 1 also displays the 20 supplements with the highest probability of appearing on webpages with H1N1, ranked by P_supplement_. We measured the public’s interest in a particular therapy by the two statistics F_supplement_ and P_supplement__._ These measures could be used together or alone in our context

### User Search Behavior: What Do Users Search for, and How Do Search Patterns Change Over Time?

**Figure 1:**
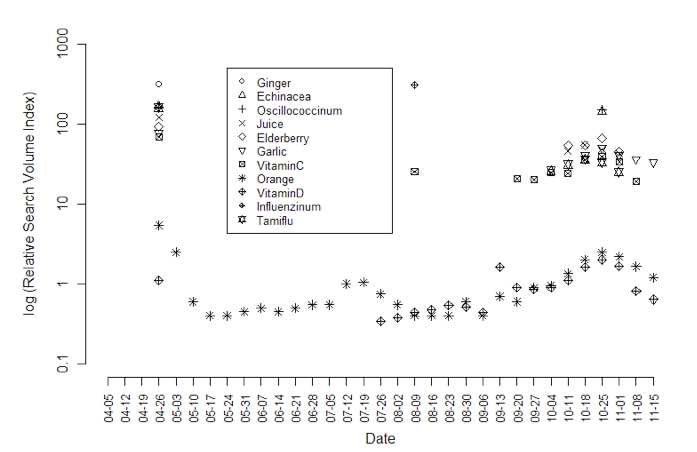
Supplement plus H1N1 queries from April 5, 2009, through November 15, 2009, as reported by Google Trends (note that only Orange had sufficient searches to appear throughout the entire period of time)

While some users contribute useful information on the Web by posting to discussion boards and blogs, others are trying to find remedies and prevention techniques in the wealth of information provided by government agencies, news organizations, and users. Figure 1 shows the temporal patterns of queries on Google for the top 10 supplements ranked by the highest relative increase in search activity during the period of the H1N1 pandemic. The baseline is the average number of searches for a supplement-plus-flu pair during the period from January 1, 2004, through December 6, 2009. The relative increase relates the peak in searches for a specific supplement during the pandemic to its baseline. Prior to April 12, 2009, very limited supplement-plus-flu search activity was reported by Google Trends; therefore, for clarity, Figure 1 displays the trends after April 12, 2009.

Figure 1 depicts search activity over time starting at the time of the first reports concerning H1N1 during the week of April 26, 2009. A precipitous drop in searches for supplement plus H1N1 occurred immediately thereafter. With the exception of searches for Orange and Vitamin D, no further significant supplement search activity (relative to all Web search activity) was detected by Google Trends until mid-September, when increasing numbers of deaths due to H1N1 were reported in the media. Supplement-plus-H1N1 search activity peaked in the last week of October, when the number of H1N1-related deaths reached 1,000. Early November 2009 is when there were reports in the news of the availability of the H1N1 vaccine. In addition to the supplements in our list, we evaluated the Federal Drug Administration’s list of banned supplements [19] for search activity associated with flu. With the exception of Airborne, the supplements on the banned list did not yield enough search activity to register in Google Trends.

### Types of Websites Providing Information About Supplements and H1N1.

The choice of query terms used for Internet searches determine the quality of information returned on the Web in terms of the websites referred to. Table 2 displays the sites listed on the first page of results from queries for H1N1 alone, supplements alone, or for supplement-plus-H1N1 pairs. The average page rank for the top 10 sites found for H1N1and for supplement-plus H1N1 query pairs were 8 and 6.4, respectively. Since higher-ranked pages are generally more authoritative, users querying for H1N1 alone arrived at sources that were more likely to be authoritative and/or actively curated and vetted by experts or organizations. For example, users were presented with the CDC website in the top slot when they queried for H1N1 alone. Table 2 demonstrates a shift from government and heavily curated sites for H1N1 alone to less authoritative or curated sites when querying for H1N1 plus supplement.

**Table 1:** Supplements with the 20 highest frequencies of occurrence (Fsupplement, left) and those with the 20 highest probabilities of co-occurrence with H1N1 (Psupplement, right).

Rank	Supplements With the Highest Frequencies of Occurrence	Frequency of Occurrence (F_supplement_)	Supplements With the Highest Probablities of Co-occurrence With H1N1	Probability of Co-occurence with H1N1 (*P*_supplement_)
1	Orange	2,570,000	Oscillococcinum	.543
2	Juice	1,450,000	Tinospora	.308
3	Vitamin D	877,000	Guduchi	.273
4	Vitamin C	827,000	Elderberry	.263
5	Onion	823,000	North American ginseng	.195
6	Green tea	649,000	Polyphenols	.116
7	Garlic	627,000	Divya giloy sat	.113
8	Ginger	581,000	Orange Juice	.105
9	Sage	427,000	Echinacea	.105
10	Rosemary	196,000	Andrographis	.103
11	Orange Juice	181,000	Ban Lan Gen	.098
12	Vitamin A	147,000	Flavonoids	.077
13	Selenium	116,000	Vitamin D	.076
14	Vitamin E	108,000	Dulcamara	.074
15	Peppermint	93,800	Elecampane	.069
16	Oscillococcinum	84,700	Jaggery	.064
17	Echinacea	82,000	Selenium	.064
18	Dulcamara	79,900	Mullein	.063
19	Elderberry	70,300	Eupatorium perforliatum	.061
20	Sulphur	68,900	Peppermint	.060

**Table 2:** Top 10 sources of information ranked by frequency of appearance on the first page of query results for H1N1 alone, each supplement alone, and each supplement plus H1N1

Rank	H1N1	Supplement	Supplement Plus H1N1
1	www.cdc.gov	en.wikipedia.org	ezinearticles.com
2	www.cdc.gov	images.google.com	hubpages.com
3	en.wikipedia.org	www.google.com	www.wellsphere.com
4	www.who.int	www.botanical.com	www.tcmwell.com
5	www.reuters.com	abchomeopathy.com	www.articlesbase.com
6	www.flu.gov	plants.usda.gov	www.ehow.com
7	news.yahoo.com	www.herbs2000.com	preventdisease.com
8	news.google.com	www.umm.edu	abchomeopathy.com
9	www.nlm.nih.gov	www.nlm.nih.gov	www.asiaone.com
10	www.cnn.com	www.elixirs.com	www.flutrackers.com

**Figure 2:**
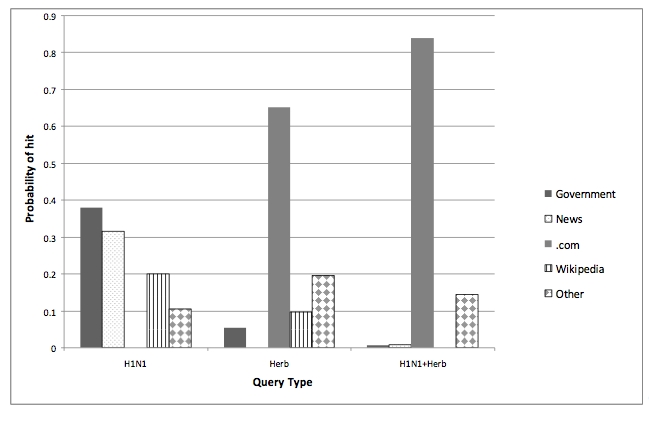
Probability distributions of hits by domain or site type for each of the three query types (distributions are based on the first page of search results)

We next calculated the proportion of hits that were from government, news, or other site types in the top 10 pages returned for all queries when querying for H1N1 alone, a supplement alone, or a supplement plus H1N1 (Figure 2). Of particular note is the change in site type proportion as the search term was changed from H1N1 alone to supplement alone to H1N1 plus supplement. The probability of arriving at a government website was .38 when querying for H1N1 alone, .05 when querying supplements alone, and only .006 when querying for supplement-plus-H1N1 pairs. When we combined supplements and H1N1 in the queries, no government entities appeared in the top 20 sites ranked by frequency of occurrence across all supplements in our sample.

### Entropy of Query Results.

In addition to being of higher quality, the results of the query are also more stable as indicated by the entropy. When we queried for the 145 supplement-plus-H1N1 pairs, it was possible to return up to 145 different sites in the top slot of the first page of query results (for comparison, we performed 145 queries for H1N1 alone). If we calculated the entropy of the set of websites that are listed in the top slot when we queried for supplement plus H1N1 pairs, the result was an entropy of 0.83 as opposed to 0.51 when we queried for the supplements alone. When we queried for H1N1 alone, the top hit was always either CDC or Google News (news.google.com) resulting in an entropy of only 0.14.

### Side Effect and Interaction Profiles of Commonly Searched Supplements

The known side effects and interactions of the 5 most commonly searched-for supplements on Google in the context of H1N1 are presented in Table 3.

**Table 3:** The 20 most frequently searched supplements in the context of H1N1, with their known uses, side effects, and interactions

Supplement	Description	Known Common Side Effects	Known Interactions
Oscillococcinum	Diluted extractions from duck liver and heart used for influenza. Contains no measurable amount of active ingredient. Not tested in pregnant women.	Unknown	Unknown
Tinospora (guduchi)	Derived from vine and used for diabetes, high cholesterol, allergic rhinitis (hayfever), upset stomach, gout, lymphoma and other cancers, rheumatoid arthritis, hepatitis, peptic ulcer disease, fever, gonorrhea, syphilis, and to boost the immune system. Not tested in pregnant women.	May reduce blood sugar in diabetics. May aggravate autoimmune diseases.	Oral hypoglycemic agents, immunosuppressants
Elderberry preparations	Used for influenza, HIV/AIDS, and boosting the immune system. It is also used for sinus pain, back and leg pain (sciatica), nerve pain (neuralgia), and chronic fatigue syndrome. Not tested in pregnant women.	May aggravate autoimmune diseases. Uncooked berries or juice can cause nausea, vomiting, and severe diarrhea.	Immunosuppressants
North American ginseng	Used for stress, to boost the immune system, to improve digestion, and as a general tonic and stimulant. Possibly unsafe in pregnant and breastfeeding women.	May cause low blood sugar, diarrhea, itching, insomnia, headache, and nervousness. Contains ginsenosides that may interfere with some estrogen-sensitive conditions.	Monoamine oxidase inhibitors, warfarin, oral hypoglycemic agents
Polyphenols	Derived from plants and includes tannins, lignins, and flavonoids. Have antioxidant properties.	Unknown	Unknown
Divya giloy sat	Ayurvedic herb with reported antiinflammatory, antipyretic properties, and immune-boosting properties. Minimal evidence exists and not tested in pregnant women.	Unknown	Unknown
Orange juice	Food product	Risk of hyperglycemia in diabetics	Unknown
Echinacea	An herb used for infections, especially the common cold and other upper respiratory infections. May decrease inflammation and boost immune system. Some limited clinical evidence and expert opinion that it may be safe in pregnancy in normal dosages.	May cause fever, nausea, vomiting, unpleasant taste, stomach pain, diarrhea, sore throat, dry mouth, headache, numbness of the tongue, dizziness, insomnia, disorientation, and joint and muscle aches. May aggravate autoimmune diseases.	Caffeine, medications metabolized by cytochrome P450 3A4 or cytochrome P450 1A2, immunosuppressants
Andrographis	Plant frequently used for preventing and treating the common cold and flu. Abortifacient	Side effects may include loss of appetite, diarrhea, vomiting, rash, headache, runny nose, and fatigue; and high doses or long-term use may cause swollen lymph glands, serious allergic reactions, and elevations of liver enzymes. May aggravate autoimmune diseases.	Antihypertensives, immunosuppressants, anticoagulants
Ban lan gen (isatis)	An herb that may have antibacterial, antiviral, antipyretic, antiinflammatory, and cancer-fighting activity. Not tested in pregnant women.	Unknown	Unknown
Flavonoids	Derived from plants, may have antiinflammatory properties. Not tested in pregnant women.	Unknown	Drugs metabolized by cytochrome P450 1A2, P-glycoprotein substrates, and anticoagulants

Vitamin D	Vitamin used for many conditions, specifically used for boosting the immune system, preventing auto-immune diseases, and preventing cancer. Likely safe in pregnant women when used in daily amounts below 50 mcg (2000 units).	Too much vitamin D may cause weakness, fatigue, sleepiness, headache, loss of appetite, dry mouth, metallic taste, nausea, vomiting, and others.	Aluminum, calcipotriene, digoxin, diltiazem, verapamil, thiazide diuretics, cimetidine, heparin, Low molecular weight heparins.
Dulcamara	Stem from vine-like plant used for acne, itchy skin, boils, broken skin, warts, arthritis-like pain, nail bed swelling, eczema, diuretic, pain relief, and calming nervous excitement. Unsafe in pregnant women or in children.	Stem is safe, though leaves or berries are poisonous. Unsafe in children.	Unknown
Elecampane	Root from herb used for cough, asthma, bronchitis, nausea, diarrhea, worms in GI tract including hookworm, roundworm, threadworm, and whipworm. Unsafe in pregnant women.	Large amounts can cause vomiting, diarrhea, spasms, and paralysis. May cause drowsiness.	Central nervous system depressants
Jaggery	Unrefined sugar used in Ayurvedic medicine for treating lung and throat infections.	Unknown	Unknown
Selenium	Mineral used for cancer prevention, heart disease, rheumatoid arthritis, diabetes. Likely safe in pregnancy when used in low doses.	Taking high doses may cause nausea, vomiting, nail changes, loss of energy, and irritability. Poisoning from long-term use is similar to arsenic poisoning, with symptoms including hair loss, white horizontal streaking on fingernails, nail inflammation, fatigue, irritability, nausea, vomiting, garlic breath odor, and a metallic taste.	Anticoagulants including warfarin, statins, niacin, barbiturates, birth control pills, gold salts.
Mullein	Flower from plant that is used for influenza, herpes viruses, and respiratory infections. Not tested in pregnant women.	Unknown	Unknown
Eupatorium perfoliatum (boneset)	Dried leaf from plant used for cancer and bacterial infections. Is cytotoxic.	Unknown	Unknown

## Discussion

In this paper, we identified a major concern regarding the types of websites on which one would most likely find information about supplements in the context of H1N1. Our ranked set of pages based on queries that contained supplements plus H1N1 or swine flu indicate that people may not be getting information from reliable sources (Table 3). Heavily curated sites that could be considered more mainstream (eg, Yahoo.com and cnn.com) or those that are more medically authoritative (eg, cdc.gov and flu.gov) did not contain supplement information in the context of H1N1.

In this study, we described the frequency with which supplements such as herbs, vitamins, and homeopathic products were displayed and how individuals searched for such information on the Web during emergence of the H1N1 pandemic. Information about the use of supplements for H1N1 was extensive, and user search activities increased and mirrored the rise in H1N1 incidence. This information was more likely to be found on alternative medicine and general information websites (eg, ehow.com), which raises the concern that those searching for supplements in the context of H1N1 will be taken to websites that are not clinically accurate, not curated for current information relative to the pandemic, or that focus on the sale of a particular supplement. One potential solution would be for government sites to provide objective information on the lack of rigorous evidence supporting the use of supplements for illnesses like H1N1 and to provide information about the potential adverse effects of supplements.

Since the Internet plays an increasing role in both public health communications and individual health–seeking behavior, this study illustrates the need for clinicians to be aware of the type and quality of health-related information available on the Internet. Our study also suggests the challenges and opportunities for Web content providers to deliver reliable and safe information to health consumers in a pandemic.

The large amount of information about supplements as they relate to H1N1 should not be surprising. Based on the 2007 report from the CDC, US consumers paid about US $14.5 billion to purchase nonvitamin, mineral, or other natural products and spent another US $2.9 billion for homeopathic medicines [20]. What is disconcerting is the lack of evidence for the efficacy for many of these products. Another major concern is the potential safety of these products and their potential interactions with conventional medications. For example, American ginseng is known to attenuate the effect of warfarin [21]. In manually inspecting the webpages returned by our searchers, we often encountered beneficial claims for these products without any mention of potential side effects or interactions with other supplements or drugs.

Our finding of an increased search activity for supplements concomitant with the rise of H1N1 incidence was probably driven by several factors. First, fear, uncertainty, and the emergence and rapid spread of an infectious disease are likely to motivate individuals to seek information. This is evident in the apparent correlation of news reports about H1N1 with search activity for supplements and Tamiflu. (Tamiflu is an antiviral drug used for the treatment of H1N1. The search activity associated with Tamiflu can be found in Figure 1.) Second, as new vaccines and treatments are developed, concerns about their safety and efficacy may lead individuals to seek alterative and natural ways of preventing or treating the disease. A recent public opinion poll found that only 40% of adults would definitely use the H1N1 vaccine if it were available [22]. Third, the distribution of effective prevention measures such as vaccines has been unequal and problematic. For example, the availability of H1N1 vaccine in primary care clinics and practices has been low, putting at risk those who are immunosuppressed or with serious chronic illnesses. Perceived unmet needs from conventional medical systems reportedly can prompt individuals to seek alternative therapies [23]. Our findings highlight that with a pandemic like the H1N1, public health agencies should consider providing on their websites objective information about supplements in the context of specific illnesses.

In an ideal clinical setting, physicians should inquire about patients’ use of supplements and be knowledgeable about reported efficacy, safety, and potential side effects or drug interactions of common supplements. Prior research has suggested that patients who concomitantly use supplements and prescribed medications rarely disclose supplement use to their health care professionals. Such disclosure is even lower for Hispanic and Asian patients, who may have additional cultural and linguistic barriers [11]. Cultural beliefs about therapeutic benefit versus harm, prior experience with health communications, and a desire for greater control over their illness may be some of the factors that lead patients not to disclose use of supplements to their physicians [24]. Therefore, patients and health care professionals should be educated to discuss supplement use in an open and supportive way. This is particularly important among the chronically ill and elderly [11,25], where polypharmacy is highly prevalent in the context of concurrent supplement use. Because of the lack of education in supplements for physicians, curriculum content in supplement use and supplement-drug interactions need to be developed and taught in all undergraduate, postgraduate, and continuing medical education. Decision support tools on these issues need to be developed and available through electronic medical record or Internet to support quick clinical decision making for both physicians and patients.

### Limitations

We based our first findings on results from Google queries. When people submit Web queries using a search engine such as Google, the resulting links may point to websites that no longer exist. Additionally, such sites may have a spurious association with the search terms (supplement and H1N1) due to random text matches, with both terms existing on the same page without being semantically related. Furthermore, the number of hits returned is an underestimate of the true number of webpages that contain the search terms since some websites are hidden and cannot be indexed by Google.

We based our second set of results on search query trends. Search queries are limited: a minimum number of searches is needed relative to all searches before Google Trends can provide trend data for a given term. Thus, we could not look at trends for some of the supplements with a high probability of co-occurrence with H1N1 (P_Supplement_) but low frequency for the supplement alone (F_Supplement_). Other issues include the inability of Google Trends to account for queries for misspelled words. For example, we would not see all of the queries for the supplement elderberry if it was misspelled as “eldaberry.” Nonetheless, we do not think that any of these limitations influenced the direction of our results or our primary findings.

### Conclusion

Although extensive information is available on the Internet with regard to natural products and H1N1, the source and quality of such information is questionable. During the emergence of H1N1, individuals actively searched for information on supplements for H1N1, and such Internet search patterns mirrored disease prevalence. Since the Internet will likely play an increasing role in human society, its delivery of timely, reliable, and scientifically sound information is critical to effective public health for dealing with current and future pandemic illnesses such as H1N1. Public health agencies should therefore consider providing on their websites objective information about supplements in the context of specific illnesses.

## Multimedia Appendix 1

Search terms including tamaflu

## Abbreviations

CDC: Centers for Disease Control and Prevention
