# Supplementary material for: Natural Supplements for H1N1 Influenza: Retrospective Observational Infodemiology Study of Information and Search Activity on the Internet
Source: J Med Internet Res. 2011 May 10;13(2):e36. doi: 10.2196/jmir.1722 (PMC3221378; doi:10.2196/jmir.1722)
Supplement: Supplementary file 1 [file jmir_v13i2e36_app1.pdf]

Multimedia Appendix 1: 145 search terms including tamaflu

Achillea	echinacea	isatis tinctoria	pot marigold
aconitum napellus	elderberry	jaggery	prunella vulgaris
adhatoda vasica	elderberry extract	jinyinhua	pulsatilla
allium cepa	elderflower	Juice	purple coneflower
Amrita	elecampane	kali bichromicum	Reishi
anas barbariae	eleutherococcus senticosus	kali carb	rhus toxicodendron
andrographis	eupatorium	lemon balm	Roselle
Aniseed	eupatorium perforliatum	lonicera japonica	rosemary
antimonium tart	euphrasia	lugen	rosmarinus officinalis
apis mellifica	ferrum phosphoricum	lycopodium	rumex
arsenicum album	flavonoids	marjoram	sage
astragalus membranaceus	forsythia	marsh mallow	sambucus nigra
baimaogen	gancao	matricaria chamomilla	scented mayweed
Ban Lan Gen	ganoderma applanatum	Melissa officinalis	schizandra berries
banlangen	ganoderma lucidum	mercurius solubilis	selenium
baryta carbonica	garlic	mullein	siberian ginseng
basil Tea	gegen	natrum muriaticum	slippery elm
belladonna	gelsemium	neem	solidago
berry extracts	ginger	nepeta cataria	spongia
black elder flower	ginkgo	North American ginseng	St. Johns wort
blue vervain	goldenrod	nux vomica	stinging nettle
Boneset	goldenseal	okra	sulphur
Bryonia	grape seed	onion	tamaflu
calcarea carb	green tea	orange	tinospora
calcarea phosphorica	guduchi	orange Juice	tongcao
calendula	heal-all	oregano	triphala
carbo veg	heye	origanum majorana	tulsi
carotenes	honeysuckle	origanum vulgare	veratrum album
Catmint	horsetail	oscillococcinum	verbena hastata
causticum	hypericum	panax quinquefolius	vitamin A
chamomile	hyssop	peppermint	vitamin C
chamomilla	Indian lilac	phosphorus	vitamin D
codonopsis	influenzinum	phytolacca	vitamin E
divya giloy sat	inula	pine bark	willow bark
dried mustard	ipecacuanha	plantain	xiakucao
dulcamara	Isatis root	polyphenols	yarrow
			zicao
